# Prevalence of haplotype DQ2/DQ8 and celiac disease in children with type 1 diabetes

**DOI:** 10.1186/s13098-022-00897-8

**Published:** 2022-09-12

**Authors:** Agnieszka Zubkiewicz-Kucharska, Tatiana Jamer, Joanna Chrzanowska, Katarzyna Akutko, Tomasz Pytrus, Andrzej Stawarski, Anna Noczyńska

**Affiliations:** 1 Department of Pediatric Endocrinology and Diabetology for Children and Adolescents, Wroclaw Medical University, Wroclaw, Poland; 2 Department of Pediatrics, Gastroenterology and Nutrition, Wroclaw Medical University, Wroclaw, Poland

**Keywords:** Diabetes type 1, Celiac disease, Haplotype HLA-DQ2/DQ8

## Abstract

Type 1 diabetes (T1D) and celiac disease (CD) coexist very often. Identification of the human leukocyte antigen (HLA) DQ2/DQ8 can confirm the genetic predisposition to CD. Negative result of this test allows to exclude CD with a high probability. It was suggested that in individuals with higher risk of CD, including T1D patients, the implementation of genetic testing should reduce the number of patients requiring systematic immunological screening. The aim of this study was to analyze the prevalence of different haplotypes predisposing to CD in children and adolescents with previously diagnosed T1D. Material and methods: A retrospective analysis was performed on 166 T1D children (91 girls) in whom HLA DQ2/DQ8 alleles were tested. In 9.6% CD was also diagnosed. Results: In 12.7% both HLA DQ2/DQ8 were negative. In 87.3% patients HLA DQ2 and/or DQ8 was positive, including 27.7% patients with both haplotypes DQ2.5 and DQ8 positive. In all CD patients the disease predisposing alleles were positive, while none of the HLA DQ2/DQ8 negative children were diagnosed with CD. Conclusions: The prevalence of HLA DQ2.5 and the HLA DQ2.5 / HLA DQ8 configuration is higher in patients with T1D, and CD compared to children with T1D alone. The combination of HLA DQ2 and HLA DQ8 most significantly increases the risk of developing CD. The group of HLA DQ2/DQ8 negative patients with improbable CD diagnosis, is relatively small. Most of T1D patients HLA DQ2/DQ8 positive need further regular antibody assessment. In patients with T1D, who are at high risk of developing CD, genetic testing may be considered to select those who require further systematic serological evaluation. Due to its retrospective nature, the study was not registered in the database of clinical trials and the Clinical trial registration number is not available.

## Introduction

Celiac disease (CD) is a chronic autoimmune enteropathy that develops as a result of an abnormal immune response to grains containing gluten in genetically predisposed individuals [1, 2]. It affects 1% of population, more often women, usually disclosing in children, however up to 20% of cases may be diagnosed in patients over 60 years of age [3, 4]. Although the exact pathogenesis of CD is not fully understood, it is known to be a multifactorial disease. Genetic, environmental, infectious as well as metabolic factors contribute to the development of CD [4, 5].

The coexisting of CD that varies from 8–18% in first degree relatives and reaches up to 70% in identical twins confirms its genetic background [6, 7]. CD has an autosomal dominant inheritance, what was shown for the first time by McDonald et al. [8] in the sixties of the past century. A close relationship between CD and histocompatibility antigens of human leukocyte antigen (HLA) class II, which are necessary, but not sufficient for developing the disease, accounting for 35–40% of the genetic risk was found. Some other genetic polymorphisms that may affect T cell reactivity in CD are being identified [7, 9–11]. It is believed that the characteristic for CD gene arrangement is DQA1*0501/DQB1*0201 encoding a DQ2.5 protein and DQA1*03/DQB1*0302 encoding the DQ8 protein. The DQ2.5 is observed in 90–95% of patients with CD comparing with approximately 25% prevalence in general population. It is considered that CD will be revealed in not more than 4% of the HLA DQ2 positive individuals [12–15]. In almost all other patients with CD the presence of the allele DQ8 was confirmed [16]. The absence of HLA-DQ2 and/or HLA-DQ8 substantially excludes the diagnosis of CD [17]. Moreover, the risk of developing CD in individuals homozygous for DQ2.5 is approximately five times higher than in heterozygotes, with an intermediate risk for heterozygotes DQ2.5/DQ8. It seems that haplotypes DQA*0201/DQB*02 (DQ2.2) and DQA1*0102/DQB1*0602 (DQ0602) carry a low risk of CD and are more often observed in patients with its latent form [18, 19].

Type 1 diabetes (T1D) and CD are often a concomitant problem. Patients with T1D are at risk for other autoimmune diseases. CD is diagnosed in approximately 2–10% of patients with T1D. This is due to a common genetic background, as both conditions are related to the HLA DQB1*0201 and DQA1*0501 alleles. Prolonged exposure to gluten is also postulated as a trigger of autoimmune process leading to T1D; moreover, it was demonstrated in animal models that dietary modification (gluten free diet) allows to reduce the incidence of diabetes [20–25].

According to the guidelines of the European Society for Paediatric Gastroenterology, Hepatology and Nutrition (ESPGHAN), in patients with an increased risk of CD, systematic diagnostic tests for CD should be performed. Examination of total IgA and specific antibodies (anti-tissue transglutaminase 2 antibodies, TGA-IgA) is recommended as the first screening test to identify persons requiring further evaluation. By ESPGHAN, patients with T1D are at risk of developing CD [26]. Following these guidelines, the Polish Diabetes Association alongside the International Society for Pediatric and Adolescent Diabetes (ISPAD) recommend screening for CD every 1–2 years for the first 10 years of the duration of diabetes [27, 28]. It must be emphasized that undiagnosed and untreated CD in T1D patients may be associated with a worsening of metabolic control (higher hemoglobin A1c, unstable blood glucose levels, a greater risk of hypoglycemia), next to the "classical" complications associated with malabsorption [29, 30].

The evaluation of the presence of HLA-DQ2/DQ8 haplotype has a high negative predictive value in CD, i.e., a negative result of this test excludes the disease with high probability [17, 26]. In individuals who belong to at-risk groups, including T1D patients, genetic testing could be performed to select people who need further systematic serological evaluation [31].

### Aim of the study

The aim of this study was to analyze the prevalence of different haplotypes predisposing to CD in children and adolescents with previously diagnosed T1D.

## Material and methods

The study included children and adolescents with T1D, patients of the Department of Pediatric Endocrinology and Diabetology, Wroclaw, Poland—which is the only center in Lower Silesia that provides care for pediatric patients with T1D. After informed consent for the genetic testing was obtained from legal guardians of all patients and all patients that are > 16 years of age, according to the Polish law, 3–5 ml of whole venous blood were collected from patients. The HLA-DQ2DQ8 determination was performed in a laboratory experienced in performing such tests. The research was conducted with the EUROArray HLA-DQ2/DQ8 test, which is used to determine the HLA-DQA1 and HLA-DQB2 alleles using special PCR primers (Table 1). This method allows you to resign from agarose gel electrophoresis, so there is no need for subjective evaluation of the test result. Samples were taken together with other routinely performed laboratory tests. Serological screening for CD is performed at the T1D diagnosis and annually in every patient.

**Table 1:** Analyzed alleles predisposing to celiac disease

Allele:	Haplotype:
HLA DQ2.2	DQA1*02:01, -DQB1*02:02
HLA DQ2.5	DQA1*05:01, -DQB1*02:01 or DQA1*05:05, -DQB1*02:02
HLA DQ8	DQA1*03:01, -DQB1*03:02 or DQA1*03:02, -DQB1*03:02

Subsequently, genetic testing was discontinued in all children with T1D, as preliminary analysis showed that waiving the annual serological screening for CD is only possible in 12% of patients, therefore genetic testing in each patient did not appear to be economically profitable.

In our Center routine serological screening for CD is performed in every patient at the T1D diagnosis and then annually.

CD was diagnosed according to ESPGHAN 2012 guidelines [32]. The diagnosis was based on the detection of elevated titers of specific antibodies: anti-tissue transglutaminase 2 antibodies (TGA-IgA) and anti-endomysial antibodies (EMA-IgA), determination of HLA DQ2 and DQ8 and esophagogastroduodenoscopy with histopathological duodenal biopsy.

### Statistical analysis

Data were presented as mean (SD, ranges) for continuous variables, and as number (%) for categorical variables. Differences between two independent groups were tested with Student’s t-test or the Mann-Whitney U-test, according to the normality of variables. To compare the allele frequencies in the different groups Fisher's exact test and Chi^2^ test were used. Binary logistic regression analysis was used to calculate the odds ratios (OR) and 95% confidence interval (CI). A two-tailed p value < 0.05 was considered statistically significant. The analysis was performed with Statistica v. 13 (TIBCO Software Inc. (2017)). Statistica (data analysis software system), Statistica version 13. http://statistica.io.Statistica v.10 (StatSoft, Inc. (2011) data analysis software system).

## Results

The study comprised 166 consecutive patients (91 girls and 75 boys) from the cohort of 1000 children treated in our Centre due to T1D, who underwent annual routine laboratory follow-up according to the recommendations by Polish Diabetes Association and the International Society for Pediatric and Adolescent Diabetes or were hospitalized due to newly diagnosed T1D, in whom the HLA DQ2 / DQ8 test was performed as part of the diagnosis of celiac disease.

The presence of the haplotype predisposing to CD was found in 146 (87.9%) of children with T1D. In 16 (9.6%) children (7 girls and 9 boys), CD was diagnosed in addition to T1D. In three cases, CD was diagnosed at the T1D presentation, whereas the remaining were diagnosed later, with the annual screening. Elevated TGA-IgA and EMA-IgA titers were found in all 16 patients. In 13/16 cases also gastroscopy and histopathological duodenal biopsies were performed, and the result of this examination confirmed CD diagnosis. In our cohort, there were no children with elevated TGA-IgA and EMA-IgA titers, who were not diagnosed with CD. All children were asymptomatic when CD was diagnosed. The mean age of the patients with T1D and CD was comparable to T1D ones (Table 2). Anthropometric parameters were comparable in children with a dual diagnosis of T1D and CD and in children with T1D only (Table 3).

**Table 2:** Age-related analysis of the studied group

	Whole group
	Mean (SD, min–max)
	N = 166
Age [years]	11.2 (4.5, 2.0–18.0)

	Males	Females	P
	Mean (SD, min–max)		
	N = 75	N = 91	
Age [years]	11.1 (4.5, 2.0–18.0)	11.2 (4.5, 2.0–18.0)	0.8723

	T1D	T1D + CD	P
	Mean (SD, min–max)		
	N = 150	N = 16	
Age [years]	11.4 (4.4, 2.0–18.0)	9.1 (4.9, 3.0–17.0)	0.0547

*T1D* Type 1 diabetes; *CD* Celiac disease

**Table 3:** Anthropometric measurements of studied patients

	Whole group
	Mean (SD, min–max)
	N = 166
Weight [kg]	38.35 (20.38, 11.25–96.50)
BMI [kg/m^2^]	18.44 (3.62, 13.18–31.48)
BMI z-score	0.32 (0.90, − 2.97–3.81)
Height [m]	1.381 (0.261, 0.875–1.993)
Height z-score	0.13 (1.05, − 2.13–3.39)

	Males	Females	p
	Mean (SD, min–max)		
	N = 75	N = 91	
Weight [kg]	38.43 (21.69, 13.00–96.50)	38.29 (19.50, 11.25–85.40)	0.7966
BMI [kg/m^2^]	18.26 (3.22, 13.18–27.96)	18.58 (3.91, 14.33–31.48)	0.9913
BMI z-score	0.30 (1.02, − 2.97–3.81)	0.28 (0.81, − 1.42–2.16)	0.9848
Height [m]	1.385 (0.279, 0.920–1.993)	1.377 (0.248, 0.875–1.793)	0.8577
Height z-score	0.22 (1.10, − 1.79–3.39)	0.06 (1.01, − 2.13–2.66)	0.4121

	T1D	T1D + CD	P
	Mean (SD, min–max)		
	N = 150	N = 16	
Weight [kg]	38.89 (19.84, 11.25–96.5)	34.31 (24.48, 13.0–85.4)	0.1431
BMI [kg/m^2^]	18.50 (3.42, 17.92–31.48)	17.98 (5.08, 13,18–30.16)	0.1463
BMI z-score	0.33 (0.87, 1.45–3.82)	0.25 (1.20, − 2.97–1.70)	0.7603
Height [m]	1.394 (0.257, 0.875–1.993)	1.268 (0.276, 0.920–1.760)	0.8822
Height z-score	0.13 (1.05, − 2.13–3.39)	0.17 (1.10, − 1.81–2.31)	0.0986

*T1D* Type 1 diabetes; *CD* Celiac disease

**Fig. 1:**
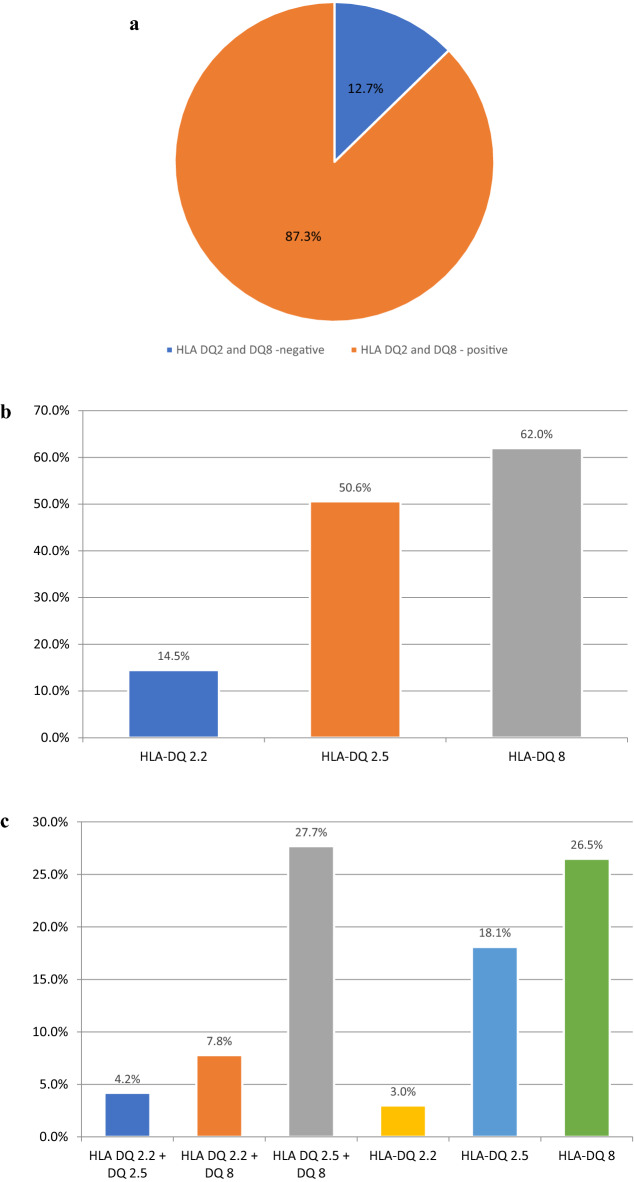
**a** Prevalence of celiac disease predisposing haplotypes in children with type 1 diabetes. **b** Prevalence of different celiac disease predisposing alleles in children with type 1 diabetes. **c** Prevalence of different configurations of celiac disease predisposing haplotypes in children with type 1 diabetes

In the whole studied group HLA DQ2.5 occurred in 50.6% of them, HLA DQ8 in 62.0% and HLA DQ2.2 in 14.5%. In 66 (39.8%) patients two alleles were found, of which the most common (46/66 patients, 27.7%) was the coexistence of HLA DQ2.5 and HLA DQ8. The prevalence of different configurations of haplotypes in children with T1D – carriers of genes predisposing to CD was shown in Fig. 1. The data on the prevalence of different configurations of haplotypes in T1D patients without CD is presented in Table 4.

**Table 4:** Prevalence of celiac disease predisposing alleles and their configurations in Lower Silesia children with type 1 diabetes

Allele:	I	II	P	OR (95% CI)
	T1D	T1D + CD		
	N = 150	N = 16		
HLA DQ2.2	23 (15.3%)	3 (18,8%)	0.5283	1.27 (0.34–4.84)
HLA DQ2.5	75 (50.0%)	13 (81,3%)	0.0220	1.0 (0.43–2.30)
HLA DQ8	93 (62.0%)	13 (81,3%)	0.0921	0.61 (0.27–1.41)
HLA DQ2.2/DQ2.5	6 (4.0%)	2 (12.5%)	0.4355	3.43 (0.63–18.61)
HLA DQ2.2/DQ8	12 (8.0%)	1 (6.25%)	0.5980	0.77 (0.09–6.31)
HLA DQ2.5/DQ8	36 (24.0%)	10 (62.5%)	0.0045	5.28 (1.79–15.53)
HLA DQ2.2 only	5 (3.3%)	0	0.6665	0.80 (0.04–15.16)
HLA DQ2.5 only	29 (19.3%)	1 (6,3%)	0.3061	0.28 (0.03–2.19)
HLA DQ8 only	42 (28.0%)	2 (12,5%)	0.1257	0.37 (0.08–1.67)
HLA DQ2/DQ8 negative	20 (13.3%)	0	< 0.0001	0.19 (0.11–3.34)

**Fig. 2:**
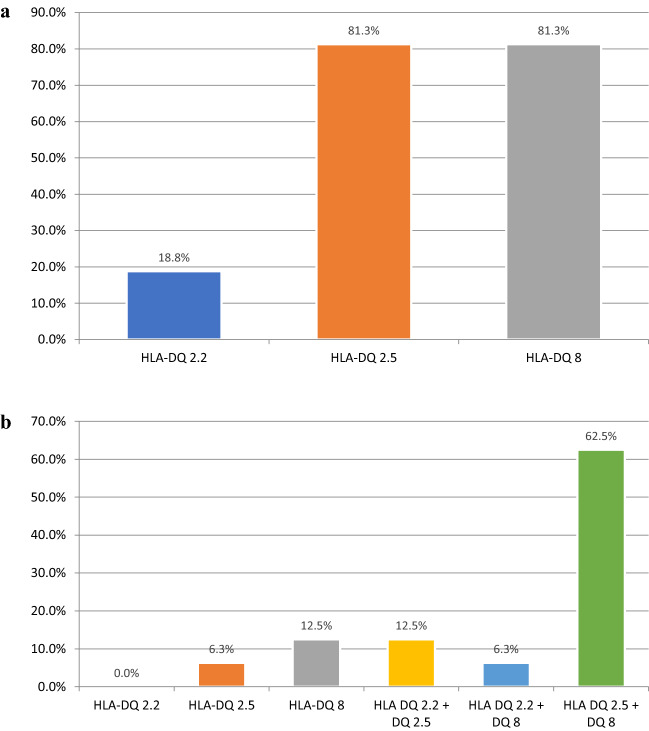
**a**. Prevalence of different celiac disease predisposing alleles in children with type 1 diabetes and celiac disease. **b**. Prevalence of different configurations of celiac disease predisposing haplotypes in children with type 1 diabetes and celiac disease

All patients with the double diagnosis of T1D and CD were carriers of genes predisposing to CD. In 13/16 two alleles were present, with HLA DQ2.5 together with HLA DQ8 as the most often (10/16) configuration. HLA DQ2.2 together with HLA DQ2.5 was present in two patients, while HLA DQ2.2 together with HLA DQ8 in only 1 patient. Moreover, HLA DQ2.5 only was carried by one patient, and HLA DQ8 by two children. The results are presented in Fig. 2.

**Fig. 3:**
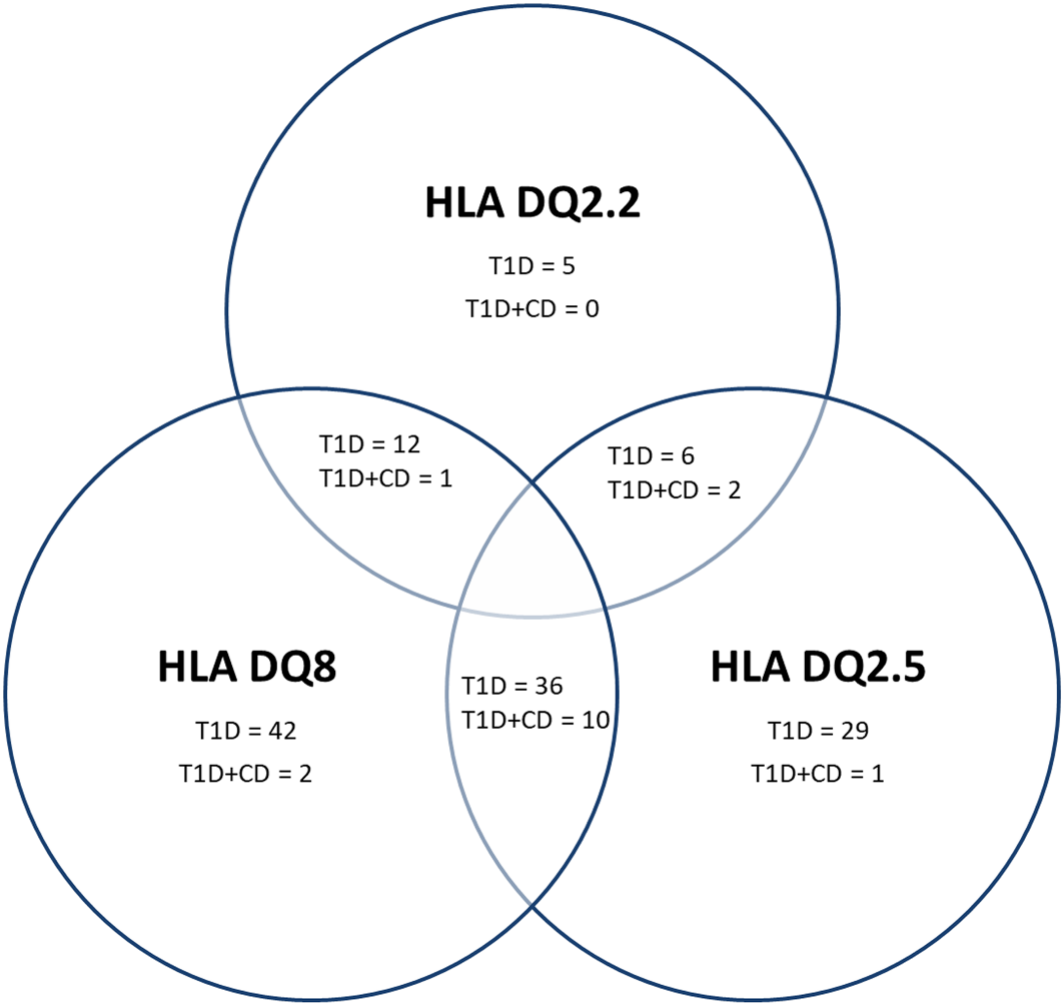
Celiac disease predisposing haplotypes in type 1 diabetes patients. T1D – type 1 diabetes, CD—Celiac disease

The prevalence of HLA DQ2.5 allele and the configuration of HLA DQ2.5 and HLA DQ8 were higher in patients with T1D and CD in comparison to children with T1D only (p = 0.0220 and p = 0.0045, respectively). The combination of HLA DQ2.5 and HLA DQ8 most significantly increases the risk of developing CD. The prevalence of other alleles predisposing to CD and their different configurations were comparable in the studied groups of children (Table 4, Fig. 3).

## Discussion

T1D is one of the most common chronic endocrine diseases that reveals itself childhood. It has an autoimmune etiology and can occur as a separate disease, but also as a component of autoimmune processes involving multiple endocrine organs (*Autoimmune Polyglandular Syndrome*, APS), most commonly co-existing with autoimmune Hashimoto's thyroiditis and CD [31, 33].

T1D, CD and autoimmune thyroiditis are similar in etiopathogenesis, that results from a common genetic background and an abnormal immune response to self-antigens [34]. It is worth noting that any delay in the diagnosis of other autoimmune diseases accompanying T1D may be a cause not only of metabolic decompensation of diabetes, but also may accelerate the occurrence of chronic microvascular complications [29, 30, 35, 36].

The prevalence of CD in patients with T1D varies from 2 to 10%, comparing to the reported prevalence of 0.5–1% of the general population [37, 38]. In the presented study, CD coexisted with T1D in 9.6% of children. Related results were obtained not only in Polish, but also in European and American evaluations. According to SWEET registry comprising 57 375 T1D patients, CD was present in 4.5% of them, more often among females. Its prevalence differs among regions, being the lowest in Asia and Middle East (1.9%) and reaching 6.9% in Australia and New Zealand [39]. In Polish population, Głowińska-Olszewska et al. [40] shown that CD was confirmed in 5.3% children with T1D diagnosed from 2010 to 2018, moreover its prevalence increased in the study period from 4.2 to 9.8%. In European countries, the co-existence of T1D and CD was most commonly observed in Denmark and Sweden (10.4% and 9.67% respectively), and least often in Portugal (2.5%), Spain (3.02%) and Greece (3.37%) [41]. This discrepancy may be explained by lower prevalence of HLA haplotypes DR3 and HLA DQB1 * 0201 in these populations [42].

It should be noted, however, that the currently obtained results indicate that "double" diagnosis of T1D and CD is significantly more frequent in children with T1D from the region of Lower Silesia than in previous years. The survey from our center from years 2006–2009 revealed that CD was diagnosed in 3.9% diabetic patients, including in 1.7% of children with newly disclosed T1D and 5.7% of children with longer-lasting diabetes [43]. On the other hand, Szalecki et al. [44] have confirmed the combination of these diseases in 2.5% of children with newly diagnosed T1D. This difference may be due to the increasing incidence of CD in the general population, as well as the shift of the peak incidence of diabetes towards younger children, and thus – with a greater risk of developing CD [45, 46]. CD most commonly is presented in the third-fourth year of diabetes duration, and the onset of diabetes earlier in life is mentioned among the factors increasing the risk of CD [47]. In our study group patients with a dual diagnosis of diabetes and CD were the age as the patients with T1D only, also age of diabetes diagnosis was similar (data not shown). Cerrutti et al. [47] concluded that a risk of developing CD is three times higher in children in whom diabetes presented before 4 years of age, compared to those who were diagnosed after 9 years of age. This was not confirmed in our study, which may also result from a small number of children examined. In the analyzed group 4/16 children with dual diagnosis were diagnosed before 4 years of age with T1D and 5/16 of these patients had developed diabetes after 9 years of age. In addition, in three cases celiac disease was diagnosed together with T1D.

The increased incidence of CD in T1D patients is due to a common genetic background comprising the HLA genotype DR3-DQ2 [21, 48]. The prevalence of HLA DQ2 is estimated at 90–95% of patients with CD, 55% of patients with diabetes and about 20–30% of the general population [49, 50]. The results obtained in our study correspond with this data. In the analyzed group, the HLA DQ2 haplotype was present in 60.8% of all surveyed children, including in seven of them (4.2%) that showed the presence of both variants of this gene: HLA DQ2.2/DQ2.5. In the group of children with a dual diagnosis of T1D and CD, the dominant HLA haplotype was DQ2 which occurred in 14/16 (87.5%) of children. HLA DQ2.5 was found in 13/16 (81.3%) of patients and in two children both HLA DQ2.2 and DQ2.5 were found. In the same number of children (13/16, 83.3%) T1D and CD, HLA DQ8 was revealed.

Approximately 90% of patients with CD present HLA-DQ2 heterodimers (HLA DQ2.5), inherited together on the same chromosome or separately on two homologous chromosomes (*cis* or *trans* configuration, respectively) [13]. Megiorni et al. [51] in their study have found that the risk of developing CD is associated with a specific HLA-DQ status. It was confirmed that the presence of DQ2 and DQ8 dimers increased the likelihood of developing CD in each case. However, differences in the incidence of disease have been demonstrated for patients with one or two copies of the predisposing DQB1 alleles. Gliadin epitopes bind to receptors on antigen presenting cells (APC) in both HLA DQ2.2 and DQ2.5 patients. However, it has been shown that in HLA DQ2.5 individuals, binding of the APC receptor to gliadin is more stable, and therefore it is presented to T lymphocytes for a longer period. This explains the higher incidence of CD in HLA DQ2.5 patients in comparison to HLA DQ2.2 patients [52]. Moreover, these antigens show immune responses to various epitopes within gliadin. The coexistence of both HLA DQ2 variants enables the presentation of more gliadin epitopes to T lymphocytes, and thus a stronger immune response, the increased risk of CD and a more aggressive course [13, 53].

Another allele predisposing to CD, HLA DQ8, also carries the risk of developing T1D. Its prevalence in patients with CD is estimated at 5–10% which that is comparable to the general population. Nearly all HLA DQ2.5 negative patients carry HLA DQ8 heterodimers [13]. In our analysis, the HLA DQ8 antigen was the most frequently found, occurring in 62% of patients. Eighty percent of patients with a dual diagnosis had the HLA DQ8 haplotype, including 70% of these children where its coexistence with HLA DQ2. For comparison, in the Danish study the presence of this haplotype was demonstrated in 60% of patients with T1D and CD, including 36% of patients with HLA DQ8 only, and in another 24% of coexistence of this allele with the HLA DQ2 [49]. In this study, however, the assessment of the haplotype was performed only in patients with CD confirmed by high antibody titer and a histopathological examination. The discrepancy between our analysis and the study by Hansen et al. [49] is most likely due to a smaller group of children with a dual diagnosis in the population we have examined.

Only about 1% of people with CD do not have HLA DQ2 or DQ8, therefore it is believed that the absence of HLA DQ2 or HLA DQ8 antigens virtually excludes the diagnosis of CD [26, 31]. In HLA DQ2/DQ8-negative CD patients, false negative results should be excluded, which may result from insufficiently detailed examination aimed at identifying less frequent alleles predisposing to CD. If the results of genetic tests (HLA DQ2/DQ8- negative) do not correlate with the results of histopathological tests of the duodenal biopsies (positive for CD), it indicates the necessity of re-evaluation of the pathologist at the reference center in order to verify the result and exclude the initially false positive result [26]. All our patients with a dual diagnosis show the presence of HLA DQ2 and/or DQ8 alleles. In the already cited study by Hansen et al. [49], in one patient (out of thirty-three subjects) with a histopathological confirmation of the diagnosis of CD, none of the predisposing alleles were found. Most studies indicate that patients with CD without genetic markers, however, meet the diagnostic criteria for irritable bowel syndrome. It is assumed that in these patients atrophy of intestinal villi is rather associated with factors other than gluten toxicity, e.g. recurrent diarrhea, and the initial diagnosis was incorrect [50]. It should be noted that none of our patients had the classic symptoms of CD, while the symptoms reported by the parents were constipation, recurrent “unexplained” hypoglycemia episodes and deterioration in glycemic control. Our observations are consistent with reports in the literature-the authors of the current ESPGHAN guidelines for the diagnosis of CD point out that the occurrence of constipation in the course of CD is reported almost as often as diarrhea [26]. According to the APC classification (age at diagnosis, presentation, complications) the typical symptoms of celiac disease include chronic diarrhea, malnutrition or malabsorption, failure to thrive and short stature, therefore constipation and poor glycemic control were not considered to be symptoms of classic celiac disease [54]. It is important to point out that the classical CD classification is being questioned nowadays. A review by Caio et al. [37] shows that symptoms considered ‘classic’ occurred only in approximately 27% of patients with CD. The authors proposed therefore the following classification: gastrointestinal, extraintestinal, subclinical, potential, seronegative, non-responsive, and refractory, however the symptoms may coexist. Furthermore, it should be emphasized that currently there is no single division of celiac disease, therefore constipation, as well as deterioration of metabolic control in concomitant T1D, may be considered non-classical symptoms of CD [37]. Metabolic control and anthropometric parameters were comparable in children with a dual diagnosis of T1D and CD to the group of children with T1D only.

The main limitation of the study is the small number of individuals surveyed and its retrospective nature. The original idea was to genetically screen all patients with diabetes that are treated in our Center, in order to identify those who will not be screened for CD in the following years due to the negative haplotype. We presented in our paper the first 166 patients (out of the 1000 cohort) genetically tested for CD-predisposing haplotype. Afterwards the genetic testing of the population was discontinued because the analysis showed that waiving the annual serological CD-screening is only possible in 12% of patients, and therefore performing the genetic test in every patient seemed to be economically unprofitable, however it has not been precisely calculated. In our study, HLA-DQ2DQ8 determination was performed using PCR-based method, considered complicated and expensive, which greatly limited its applicability as a population screening for CD genetic predisposition. Monsuur et al. [55] presented a highly accurate method of HLA allele determination based on Tag Single Nucleotide Polymorphisms (SNPs), proving that testing only 6 SNPs is has a sensitivity of > 0.991, specificity > 0.996, and a predictive value > 0.948. Such method is also much cheaper comparing to the one we have used and provides an excellent basis for population screening for CD. Unfortunately, our study is also too small to perform the haplotype distribution. Obviously, as T1D and CD share HLA genes, especially the alleles for DQ8, the search for more alleles of type DQ2 and DQ7 might allow to expand the negative predictive profile of the sample [55].

In conclusions, it should be stated that patients with T1D are at greater risk of developing other autoimmune diseases [26]. CD is one of the most common autoimmune diseases associated with T1D [56]. Delaying the diagnosis of CD may adversely affect the course of diabetes. Therefore, it is reasonable to perform systematic screenings for the most common comorbid autoimmune diseases, including CD [26]. In questionable cases of CD with discrepant serology and histopathology results, HLA testing may be a useful tool identify the individuals who are at-risk of developing CD and those without genetic predisposition to develop CD. The prevalence of HLA DQ2.5 and the HLA DQ2.5 / HLA DQ8 configuration is higher in patients with T1D, and CD compared to children with T1D alone (p = 0.0220 and p = 0.0045, respectively). The combination of HLA DQ2 and HLA DQ8 most significantly increases the risk of developing CD. Furthermore, it may be used as a screening test for first-degree relatives of CD or T1D patients, as the incidence of CD among them is higher. HLA DQ2/DQ8 positive individuals would require regular clinical and serological monitoring. Moreover, HLA typing may be considered in genetic counseling to determine the risk of disease [57, 58]. Unfortunately, due to shared genetic predisposition, the group of patients with T1D who will not need routine systematic immunological testing for CD is small. In the remaining cases—in which the HLA DQ2 / DQ8 result is positive—they will require further regular monitoring for the presence of antibodies, especially since most cases are asymptomatic. Therefore, routine HLA typing tests in patients with T1D seems to be unprofitable. However, this requires detailed analyzations based on clinical trials on large groups of patients [26].

## Data Availability

The data presented in this study are available on request from the corresponding author. The data are not publicly available due to the content of personal data of patients.
